# Silicon Modifies Photosynthesis Efficiency and *hsp* Gene Expression in European Beech (*Fagus sylvatica*) Seedlings Exposed to Drought Stress

**DOI:** 10.3390/genes15091233

**Published:** 2024-09-21

**Authors:** Justyna Nowakowska, Monika Dang, Piotr Kiełtyk, Marzena Niemczyk, Tadeusz Malewski, Wiesław Szulc, Beata Rutkowska, Piotr Borowik, Tomasz Oszako

**Affiliations:** 1 Faculty of Biology and Environmental Sciences, Institute of Biological Sciences, Cardinal Stefan Wyszynski University in Warsaw, 01-938 Warsaw, Poland; j.nowakowska@uksw.edu.pl (J.N.); p.kieltyk@uksw.edu.pl (P.K.); 2 Department of Silviculture and Forest Tree Genetics, Forest Research Institute, 05-090 Sękocin Stary, Poland; m.niemczyk@ibles.waw.pl; 3 Department of Molecular and Biometric Techniques, Museum and Institute of Zoology, 00-679 Warsaw, Poland; tmalewski@miiz.waw.pl; 4 Institute of Agriculture, Warsaw University of Life Sciences, 02-787 Warsaw, Poland; wieslaw_szulc@sggw.edu.pl (W.S.); beata_rutkowska@sggw.edu.pl (B.R.); 5 Forest Protection Department, Forest Research Institute, 05-090 Sękocin Stary, Poland; pborow@poczta.onet.pl

**Keywords:** abiotic stress, water stress drought, maximum efficiency of PSII, Si fertilizer

## Abstract

**Background:** Climate change is leading to severe and long-term droughts in European forest ecosystems. can have profound effects on various physiological processes, including photosynthesis, gene expression patterns, and nutrient uptake at the developmental stage of young trees. **Objectives:** Our study aimed to test the hypothesis that the application of silica (SiO_2_) influences photosynthetic efficiency and gene expression in 1- to 2-year-old *Fagus sylvatica* (L.) seedlings. Additionally, we aimed to assess whether silicon application positively influences the structural properties of leaves and roots. To determine whether the plant physiological responses are genotype-specific, seedlings of four geographically different provenances were subjected to a one-year evaluation under greenhouse conditions. **Methods:** We used the Kruskal–Wallis test followed by Wilcoxon’s test to evaluate the differences in silicon content and ANOVA followed by Tukey’s test to evaluate the physiological responses of seedlings depending on treatment and provenance. **Results:** Our results showed a significantly higher Si content in the roots compared with the leaves, regardless of provenance and treatment. The most significant differences in photosynthetic performance were found in trees exposed to Si treatment, but the physiological responses were generally nuanced and provenance-dependent. Expression of *hsp*70 and *hsp*90 was also increased in leaf tissues of all provenances. These results provide practical insights that Si can improve the overall health and resilience of beech seedlings in nursery and forest ecosystems, with possible differences in the beneficial role of silicon application arising from the large differences in wild populations of forest tree species.

## 1. Introduction

Intense summer droughts, heat waves, and changing rainfall patterns are considered major threats from global climate change. These factors weaken tree vitality, reduce biomass production [1], and destabilize forest ecosystems and water and carbon cycles [2,3,4].

European beech (*Fagus sylvatica* L.), one of the most important forest-forming tree species in Europe, is sensitive to drought, which makes it very vulnerable to climate change-induced heatwaves and droughts [5,6]. A significant decline in beech growth is expected in large parts of its range [7,8]. Therefore, the development of strategies to improve the drought tolerance of this important tree species is urgently needed. One promising solution lies in the use of silicon, a microelement that makes up 27.7% of the Earth’s lithosphere.

Silicon is abundant in silicate minerals such as quartz (SiO_2_) and feldspar (NH_4_AlSi_3_O_8_), as well as in soluble forms like silicic acid ions (SiO_2+*n*_)^2*n*−^, which higher plants preferentially absorb from the soil. Once taken up by roots, soluble silicon is primarily deposited in plant tissues as phytoliths, contributing to increased stress tolerance, including resistance to drought [9]. The application of exogenous silicon improves silicon uptake and accumulation in plants and facilitates the absorption of important nutrients such as nitrogen, phosphorus, and calcium. This process leads to a thickening of the leaves and stems, reflected in a higher average weight and a remarkable increase in the chlorophyll concentration in the leaves [10,11]. It also influences the arrangement of the leaves and has a positive effect on the absorption of light energy. This increases the photosynthetic efficiency of various plants such as rice, wheat, sugar cane, bananas, cucumbers, and amaranth [9]. The addition of silicon has been shown to promote the growth of rice roots, increase root sprouting (by 20–30%), and improve the absorption of water and nutrients from the rhizosphere [12,13].

Scientific research has extensively investigated the relationships between drought, forest tree growth, photosynthetic efficiency, and gene expression analysis. Recently, some important results have shown that Si uptake can positively influence the growth and development of oak and pine seedlings [14]. In agriculture and horticulture, the use of Si increases yields (biomass) and the quality of fruit (e.g., raspberries or strawberries), which become more durable and better able to withstand transport [15,16,17,18]. However, the most important effect of Si seems to be related to the fact that the plants manage their water resources better and survive periods of drought. The closure of the stomata under the influence of silica reduces transpiration, minimizing water loss as a water-saving mechanism under deficit conditions [19].

The chlorophyll fluorescence measurement is a very sensitive method that allows for the detection of changes in the general bioenergetic state of the plant photosynthetic apparatus of many crops and forest tree species under either biotic and abiotic stress conditions [20,21,22]. The significant variations in photosynthetic efficiency have been observed in seedlings of *Betula pendula* (Roth.) subjected to the pathogen *Phytophthora plurivora* (T. Jung and T.I. Burgess) infection [23] and in leaves of *Fagus sylvatica* (L.) under drought [24].

Drought often leads to the oxidative stress in the plant cells, which triggers up- or down-regulation of many stress-responding genes encoding proteins of rescue, e.g., lipoxygenases, peroxidases, and heat shock proteins (HSP) [25]. Among a large family of *hsp* genes, *hsp*70 and *hsp*90 are differentially expressed in many crops under flood [26], salinity [27], heavy metal exposure [28], and drought [29,30,31,32].

Our study aimed to test the hypothesis that silicon increases photosynthetic efficiency and induces the expression of *hsp*70 and *hsp*90 genes under drought conditions, thus helping to mitigate the negative effects of drought stress. We also wanted to find out whether the application of silicon positively influences the structural properties of leaves and roots of *Fagus sylvatica* seedlings. Ultimately, we hope that a deeper understanding of the mechanisms behind the acclimation of beech seedlings to drought, which are enhanced by foliar application of silicon, will support the development of sustainable forest management practices, especially in the context of climate change and increasing drought in forest ecosystems.

## 2. Materials and Methods

### 2.1. Plant Material, Growing Conditions, and Experimental Setup

**Figure 1:**
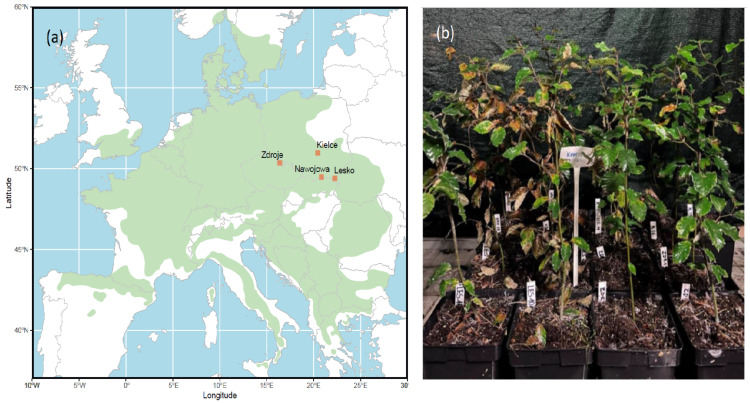
(**a**) Location of the beech provenances from which the seeds for the experiment were taken against the background of the geographical distribution of *F. sylvatica*. (**b**) Experimental setup of pots with one-year-old *F. sylvatica* from four Polish provenances grown under greenhouse conditions (photo by M. Dang).

To test whether the physiological responses of the plants depend on the genotype, four provenances of 1–2-year-old beech seedlings were tested. Therefore, before starting the experiment, 64 one-year-old beech seedlings from four different Polish provenances, namely Lesko FD (LES) (49°28′28″ N 22°19′44″ E), Nawojowa FD (NAW) (49°33′38″ N 20°44′40″ E), Kielce Forest District (KIE) (50°52′27″ N 20°38′00″ E), and Zdroje FD (ZDR) (50°26′18″ N 16°14′22″ E) (Figure 1a), were grown in 5 L pots with soil enriched with Osmocote 3M fertilizer (1.5 g/L). Such a preparation of soil substrate contained 16% of N, 7% of P_2_O_5_, and 18% of K2O, with traces of Mg, Fe, Zn, and Cu.

From July 2022 to June 2023, all pots were placed in a greenhouse with controlled humidity and temperature at the Forest Research Institute in Sękocin Stary, Poland (Figure 1b). Humidity was maintained at around 60%, with temperatures kept between 15 °C and 17 °C. The air temperature and humidity were measured by HOBO U23-001A (Onset, MA, USA). The lighting of the beech trees depended solely on the natural light that entered through the glass panes of the greenhouse.

The seedlings of each provenance were divided equally (i.e., four plants per provenance) into four experimental treatments consisting of the combination of Si supply and drought conditions: A—control, B—spraying of Si (OPTISIL® MICRO, Intermag S.A., Olkusz, Poland) + water, C—spraying of Si (OPTISIL® MICRO) + drought, D—drought. All plants of variants A and B were irrigated with tap water every two days. Drought conditions (C and D) were simulated by reduced irrigation every 9–10 days with 200–250 mL tap water per pot, with soil relative humidity reduced to 0.1 m^3^/m^3^ which was measured with the ECH2O Utility—Em50 sensor (Decagon Devices, Inc., Pullman, WA, USA).

Foliar spraying of plants of variants B and C was repeated 19 times every 7–10 days with OPTYSIL® diluted in a ratio of 2 mL solution in 1 L distilled water and containing 200 g SiO_2_ (16.5% m/m) according to the manufacturer’s recommendations (Intermag S.A., Olkusz, Poland).

### 2.2. Determination of Si Content in Roots and Leaves

The roots from tree trees per treatment in each provenance were carefully washed in distilled water and dried, along with the leaves from the same seedlings, which were collected and dried to a constant weight. The material was then ground in a Retsh mill at 5000 rpm.

The silicon content was determined using the ICP method (IRYS Advantage ThermoElementar, Cambridge, UK) after wet mineralization in a closed system in a microwave (Ethos Up, Milestone INC, Shelton, CT, USA) (0.3 g of the plant material was added to 10 cm^3^ of a mixture of concentrated acids HNO_3_ and HClO_4_ in a ratio of 5:2 and mineralized for 0.5 h).

### 2.3. Test of Chlorophyll Fluorescence Parameters

Chlorophyll fluorescence emitted by the photosynthetic apparatus was measured on four selected leaves from four seedlings per treatment in July 2022 and October 2022, i.e., before and after Si treatments. Leaves were selected from the bottom, middle, and upper parts of the seedlings. We ensured that only healthy leaves, free from fungal diseases, were used for fluorescence measurements, although we could not completely avoid aphid presence. All the leaves measured were mature. In all measurements, the leaf blade was placed for about 20 min in the dark (using special clips) and then excited for 1 s with a light pulse of 3500 mol/m^2^/s using a Handy PEA+ device (Hansatech Instruments Ltd., King’s Lynn, Norfolk, UK). Using this technique, the following photosynthetic parameters were determined according to Kalaji et al. [22]:

- Fo/Fm (state of electron transfer of PSII);
- Fv/Fm (maximum quantum yield of the photochemistry of PSII);
- ABS/RC (ratio of active to inactive RC, reflecting the absorbed energy flux through the active RC);
- DIo/RC (total dissipation of energy not absorbed by the RC in the form of heat, fluorescence, and transfer to other systems at t = 0);
- TRo/RC (capture of photon energy by an active RC, at t = 0);
- ETo/RC (electron transport rate through an active RC, at t = 0);
- PI Inst (performance index reflecting the instantaneous functional index of the PSII);
- PI abs (PSII function index, calculated on the basis of energy absorption);
- PI tot (overall performance index of PSII, which is inversely proportional to photosynthetic efficiency).

### 2.4. Gene Expression Analysis

To analyze the gene expressions of *hsp*70 and *hsp*90, the total RNA of 1–2 fresh beech leaves (approx. 200 mg) was crushed in a mortar in the presence of 5 mL RLK buffer of the Plant RNA MINI kit (Syngen Biotech, Wrocław, Poland) and mixed with 50 μL 2-mercaptoethanol (Sigma Aldrich, Milwaukee, WI, USA) according to the manufacturer’s instructions (Syngen Biotech, Wrocław, Poland). Two μL of the isolated RNA were used for cDNA synthesis using the NG dART RT kit (EURx, Gdańsk, Poland) according to the manufacturer’s instructions (EURx, Gdańsk, Poland). Quantitative (qPCR) analysis was performed on a ThermoFisher Scientific instrument (Waltham, MA, USA) using the dedicated qPCR SYBR Master Mix kit (Sigma Aldrich, Milwaukee, WI, USA) under the following conditions: initial denaturation at 95 °C for 3 min, then 40 cycles consisting of denaturation at 95 °C for 30 s, forward and reverse primers annealing at 60 °C for 60 s, and final extension at 72 °C for 2 min.

The primers used for the amplification of the genes *hsp*70 and *hsp*90 were: Forward 5′-AGGATTGCTCC-GACAAGGC and Reverse 5′-CAACGCCTCCATGAACTCCT. The actin gene (AM06302) served as a reference [33]. All expression analyzes were performed separately in three independent replicates for each gene and finally averaged using the program Do My qPCR [34].

### 2.5. Statistical Data Analysis

To compare silicon content in leaves and roots between the four experimental treatments, four measurements of silicon content were made per treatment, resulting in a total of 16 measurements of silicon content in leaves and 14 measurements of silicon content in roots. The four seedlings on which the silicon content was measured within each treatment came from four provenances: LES, NAW, KIE, and ZDR. Since the root silicon content data were not normally distributed, as shown by the Shapiro-Wilk test, a non-parametric Kruskal–Wallis test followed by a pairwise post-hoc Wilcoxon rank sum test was used to assess the differences in mean silicon content between the experimental treatments. The non-parametric Wilcoxon rank sum test was also used to test for differences between silicon content in leaves and roots, both within treatments and for pooled data. To test the effects of the different treatment conditions, date of measurement, and provenance on chlorophyll fluorescence parameters, a multi-factor ANOVA followed by a Tukey’s Honest Significant Difference (HSD) post hoc test was used [35]. To ensure the validity of the ANOVA, we checked the normal distribution of the ANOVA residuals with the Shapiro-Wilk test and tested the homogeneity of variances between the treatment levels. Differences in *hsp*70 and *hsp*90 gene expressions between controls and experimental treatments were assessed with t test using the Do my qPCR software [34].

The normality of the distribution was tested with the function *shapiro.test()* from the R base installation, while the homogeneity of the variances was tested with the function *leveneTest()* from the auto package [36]. The non-parametric Kruskal–Wallis test was performed with the function *kruskal.test()* and the post-hoc Wilcoxon pairwise comparison with the function *pairwise.wilcox.test()*, both functions from the basic R installation. The two-way ANOVA was performed with the function *aov()* and the Tukey HSD test with the function *TukeyHSD()*, both functions from the R base installation version 4.1.1 [37].

## 3. Results

### 3.1. Silicon Content in Leaves and Roots

**Figure 2:**
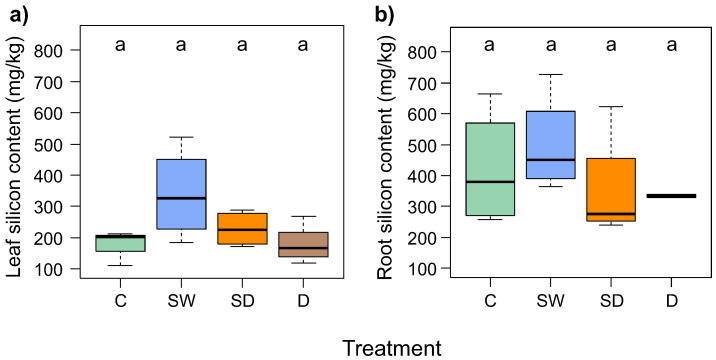
Comparison of silicon content in leaves (**a**) and roots (**b**) *F. sylvatica* seedlings subjected to experimental treatments: C—Control, SW—Spray + Water, SD—Spray + Drought, D—Drought. The letters above the boxes indicate the results of the post-hoc Wilcoxon rank sum test. Treatments that do not have a common letter are significantly different at the 0.05 significance level. The horizontal lines within the boxes represent the medians; the boxes define the interquartile range IQR (25–75%), and the whiskers extend to the IQR × 1.5 range to indicate possible outlier observations outside their range.

The silicon content in the leaves was higher in the experimental treatment Spray + Water (Figure 2a, mean = 339.5 mg/kg) than in Spray + Drought (mean = 228.5 mg/kg), Control (mean = 181.1 mg/kg), and Drought (mean = 178.9 mg/kg). However, the differences in the silicon content of the leaves between the treatments were not statistically significant (Figure 2a).

The silicon content in the roots had the highest value in the Spray + Water treatment (Figure 2b, mean = 498.8 mg/kg) than in the control (mean = 420.4 mg/kg) and Spray + Drought (mean = 354.2 mg/kg). The differences in the silicon content of the roots between the treatments were not statistically significant (Figure 2b).

The comparison of the silicon content of leaves and roots showed that the silicon content in the roots was significantly higher than in the leaves in all treatments (*p* < 0.001). However, when analyzed within treatments, the difference between silicon content in leaves and roots was statistically significant only in the control (*p* = 0.029), while there were no significant differences in Spray + Water (*p* = 0.343) and Spray + Drought (*p* = 0.343).

### 3.2. Chlorophyll Fluorescence Parameters

**Figure 3:**
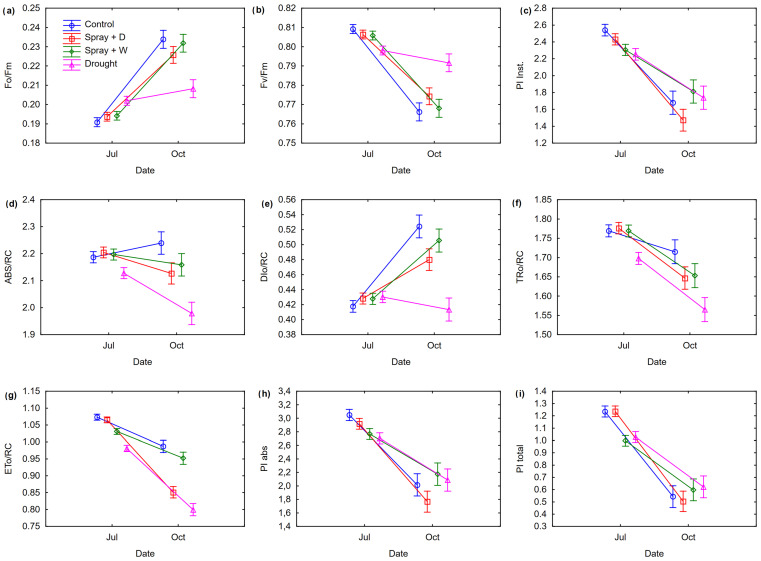
Comparison of chlorophyll fluorescence parameters of *F. sylvatica* seedlings subjected to experimental treatments: Control, Spray + W—Spray + Water, Spray + D—Spray + Drought, Drought in different dates of measurements: July and October. Vertical symbols represent standard errors.

The dates of the chlorophyll fluorescence measurements had a significant influence on the values of all parameters evaluated (Table 1). In general, fluorescence parameters were higher in July than in October (Figure 3). Treatment also had a significant effect on ABS/RC, DIo/RC, TRo/RC, and ETo/RC, with beech seedlings showing higher values in the Control, Spray + Drought, and Spray + Water treatments. The lowest photosynthetic activity of PSII was observed under drought conditions. Of note, seedlings in the drought treatment had lower Fv/Fm values than seedlings in the other treatments as early as July, indicating early signs of photoinhibition due to drought stress (Figure 3).

**Figure A1:**
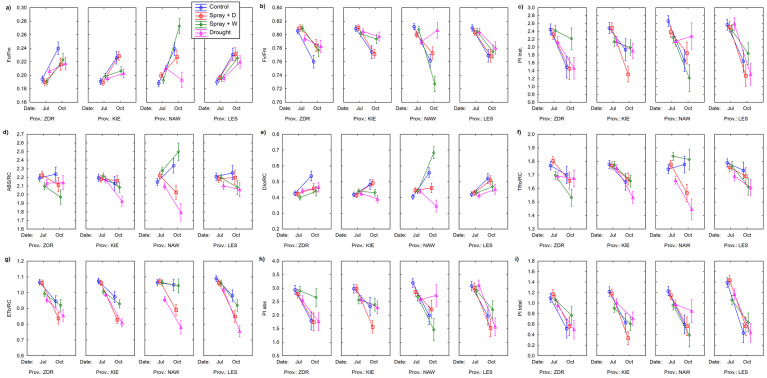
Comparison of chlorophyll fluorescence parameters of *F. sylvatica* provenances subjected to experimental treatments: Control, Spray + W—Spray + Water, Spray + D—Spray + Drought, Drought in different dates of measurements: July and October. Vertical lines represent standard errors.

ANOVA revealed provenance-specific responses of beech seedlings to treatments and measurement dates for most of the fluorescence traits examined (Table 1). Among the provenances, Nawojowa (NAW) demonstrated a specific response to the Drought and Spray + Water treatments. Figure A1 provides a detailed insight into the individual provenances’ responses to the chlorophyll fluorescence parameters. The interaction between provenance, treatment and measurement date was significant for F0/Fm, Fv/Fm, ABS/RC, and TRo/RC.

### 3.3. Different Level of *hsp* Genes Expression

**Figure 4:**
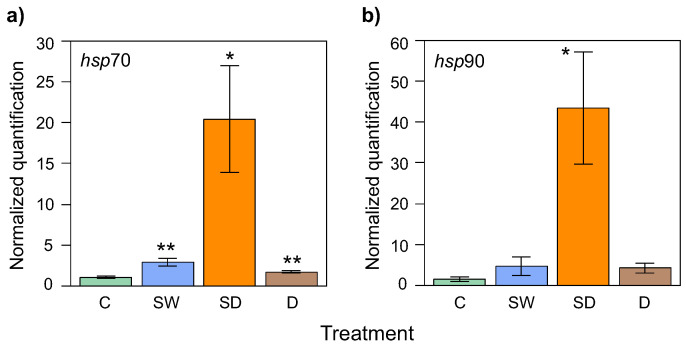
Overall expression of (**a**) *hsp*70 and (**b**) *hsp*90 genes with a standard deviation value in four experimental variants of European beech seedling treatment: C—Control, SW—Spray + Water, SD—Spray + Drought, D—Drought. Asterisks above bars indicate significant differences in t test between experimental group and the control group; *—*p*-value 0.05–0.01, **—*p*-value 0.01–0.001.

The specificity and stability of the acting gene as a reference in qPCR amplification were proved by the Ct values obtained for the selected cDNAs and confirmed by the presence of a single peak in the melting curve obtained. The highest overall expressions of *hsp*70 and *hsp*90 genes were observed for Spray + Drought treatment, reaching the level 20 of normalized quantification for *hsp*70 and 43 for gene *hsp*90 (Figure 4). Water-deficient plants supplemented with Si (variant B) and plants subjected only to drought (variant D) produced the comparable mRNA level of two studied *hsp* genes as control plants (variant A) (Figure 4a,b).

**Figure 5:**
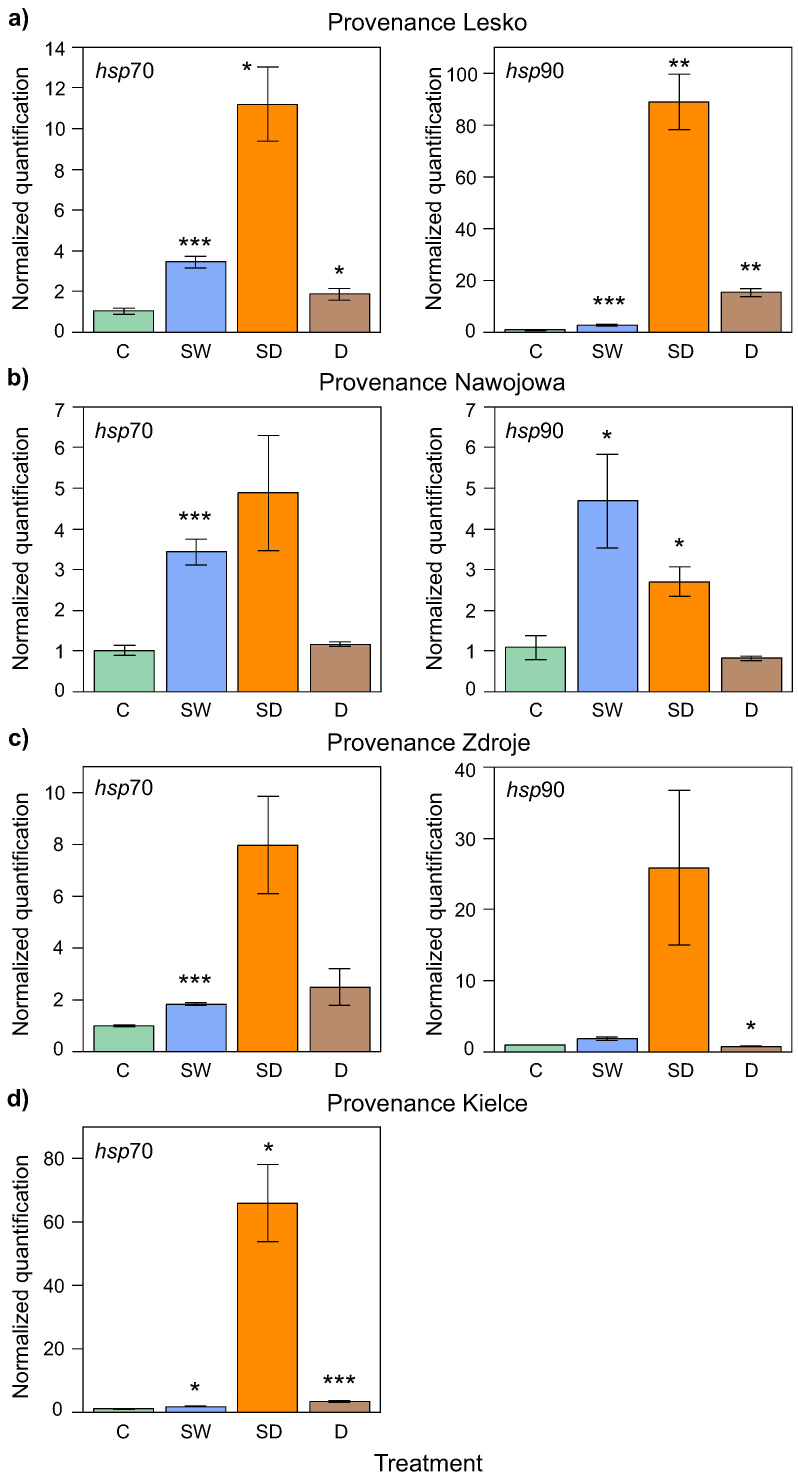
Effect of beech seedlings provenance on *hsp*70 and *hsp*90 genes relative expression in four experimental variants. (**a**) Provenance Lesko; (**b**) Provenance Nawojowa; (**c**) Provenance Zdroje; (**d**) Provenance Kielce. Treatments: S—Control, SW—Spray + Water, SD—Spray + Drought, D—Drought. Due to the limited leaf amount for Kielce provenance, only a *hsp*70 expression was measured. Asterisks above bars indicate significant differences in t test between experimental group and the control group; *—*p*-value 0.05–0.01, **—*p*-value 0.01–0.001, ***—*p*-value < 0.001.

The *hsp*70 expression was significantly up-regulated in the provenances of Lesko, Zdroje, and Kielce compared with the control (Figure 5a,c,d). The highest level (69) of positive *hsp*70 regulation was denoted for seedlings from Kielce (Figure 5d), and the lowest level (1) was for seedlings from Nawojowa (Figure 5b). Because of material shortage, not all provenances were measured for *hsp*90 expression; nevertheless, the expression of *hsp*90 was significantly higher (90) in the Spray + Drought treatment of seedlings from Lesko (Figure 5a). The same shape of up-regulation was observed for Zdroje (Figure 5c). Only plants from Nawojowa showed slightly the highest relative expression of the *hsp*90 gene (level 4.8) in regularly watered plants supplemented with Si compared with the control (1.1) (Figure 5b).

## 4. Discussion

### 4.1. Changes in PSII Activity under Drought Stress

Many studies have documented significant physiological changes in the photosynthetic responses of forest tree species in response to drought [38]. These changes include a decrease in photosynthetic rates, which are accompanied by a reduction in stomatal conductance and chlorophyll concentration. Prolonged stomatal closure, indicative of low stomatal conductance, can hinder growth and increase the tree’s susceptibility to photooxidative stress. In severe cases, prolonged stomatal closure due to drought may lead to tree mortality [39]. The potential quantum efficiency of PSII, as reflected by dark-adapted Fv/Fm values, is a sensitive indicator of a plant’s photosynthetic performance [40,41]. Optimal values for most plant species typically range around 0.83 [40,42]. Deviations from this optimal range can indicate that the plant has been subjected to stress, particularly suggesting the occurrence of photoinhibition.

In our study, the maximum quantum yield of PSII, as indicated by Fv/Fm values, hovered around 0.8 in July across most treatments, with the exception of the drought treatment, where seedlings’ Fv/Fm dipped below this threshold. By October, Fv/Fm values had decreased across all treatments, suggesting a seasonal shift in chlorophyll content and a reduced quantum efficiency of PSII—a finding consistent with other research on temperate tree species [43] and tropical species [44]. The most pronounced differences between treatments emerged in October, following several months of experimental conditions. The most significant disparities were observed between the drought treatment and the others, although the seedlings’ responses were nuanced and somewhat equivocal. The ambiguity in our chlorophyll fluorescence data are underscored by statistically significant differences that favored the control treatment (and, to a lesser extent, the Spray + Water and Spray + Drought treatments) in terms of Fo/Fm, ABS/RC, TRo/RC, and ETo/RC. This suggests that the stressed seedlings under drought conditions were in a relatively poorer condition; however, contrary to this, under the Drought condition, certain parameters diverged significantly from the control, such as Fv/Fm and DIo/RC, which might paradoxically indicate better physiological functioning of the seedlings under stress.

These seemingly conflicting results may be explained by the intricate interplay between the beech’s inherent stress tolerance mechanisms and the specific environmental conditions of the study. The beech’s anisohydric nature [45] allows it to strategically manage water loss and photosynthetic activity in response to drying conditions [46]. This adaptability might have allowed the beech seedlings to maintain relatively high PSII efficiency even under moderate drought stress, as evidenced by the higher Fv/Fm values. Nonetheless, the significantly higher values in parameters like ABS/RC, TRo/RC, and ETo/RC under the control and Spray + Water treatments, compared with those under drought conditions, could suggest that these seedlings were not entirely unaffected by the stress, as these parameters are indicative of the energy absorption and electron transport processes within the photosynthetic system, which may have been compromised by the drought conditions [47]. The higher values observed under the Drought condition for parameters such as DIo/RC could reflect a short-term acclimation response or a temporary rebalancing of light harvesting and photoprotective mechanisms. Overall, the results highlight the beech’s resilience to drought stress but also underscore the intricate and dynamic nature of its physiological responses to environmental challenges. These indicate responses are further nuanced by provenance-specific responses of beech seedlings to treatments and treatments with interaction with time (date).

The evaluation of chlorophyll a fluorescence signals in various forest tree species (European ash, European beech, horse chestnut, European oak) under biotic and abiotic stresses has emerged as an increasingly valuable technique for monitoring plant health in both greenhouse and field settings [48,49,50]. Despite its growing utility, there remains a dearth of research focusing on the impact of silicon fertilizer on the mitigation of drought injury in European forest tree species. The existing literature predominantly concentrates on tropical tree species, such as *Lacistema aggregatum* (P.J. Bergius, Rusby) and *Inga vera* (Willd. LC.) [51,52]. Recently, it has been shown that Si fertilizer increased photosynthetic efficiency (PI total), biomass, and growth of one-year-old *Pinus sylvestris* (L.) and two-year-old *Quercus robur* (L.) seedlings [14]. Moreover, the application of the Si spray in concentrations of 1% and 2% reduced the infestation of and damage to oak leaves by powdery mildew *Erysiphe alphitoides* (Griffon and Maubl.) U. Braun and S. Takam [14].

### 4.2. Drought and Silicon-Modulated Gene Expression in Plants

To investigate the very first molecular response of the plants under drought conditions and Si supply, two types of heat shock protein genes (*hsp*70 and *hsp*90) were investigated. The chloroplast *hsp*70 protein is generally involved in protein transport and development [53,54,55,56], and the cytoplasmic HSP90 plays an important role in pathogen resistance response by interacting with the proteins, which act as signaling receptors for the pathogen infection [57,58]. It has been demonstrated in *Hordeum vulgare* (L.) and *Zea mays* (L.) that some HSPs (e.g., HSP70 and sHSP26, respectively) improve chloroplast performance under drought stress by interacting with specific chloroplast proteins [59,60].

Our studies have shown that one-year-old beech seedlings respond to hydric stress and Si supply by increasing the expression of heat shock proteins (HSP70 and HSP90). Based on a detailed analysis of *hsp*70 and *hsp*90 gene expression, it can be concluded that the drought conditions and the application of the Si preparation alone are not significant factors triggering stress response reactions in European beech seedlings (or at least not to the extent that would lead to changes in the expression of the examined heat shock genes). However, the combination of these stress-inducing factors—drought and foliar Si spray—significantly increased the expression level of these genes. Particularly in the case of *hsp*90 gene expression, this level increased several dozen times in all seedlings from two origins (Lesko, Zdroje) compared with the control. In one provenance (Nawojowa) in normal watering conditions, Si alone caused a higher *hsp*90 expression than in the control plants, suggesting its impact on plant reaction to this natural fertilizer. The modulation of *hsp*70 and *hsp*90 gene expressions in beech seedlings under hydric stress conditions, and the subsequent compensation through silicon supplementation suggest a potential protective role of silicon in mitigating the impact of water stress on plants at the molecular level.

It is premature to conclude which concrete provenance better tolerates drought conditions in its early stage of growth. From the genetic point of view, our previous study of several European beech provenances based on neutral microsatellite DNA markers demonstrated that the provenances of European beech from the south (Lesko and Nawojowa in the present study) have a double-higher differentiation coefficient (Fst = 0.091) in comparison to the provenances from the west (Fst = 0.048) of Poland, like Zdroje [61]. Such a pattern of genetic variation may result from the adaptation of beech trees to local environmental conditions and human activity in recent centuries, e.g., planting from various seed sources [62,63,64].

Recent research indicates that silicon may play a role in regulating protein-coding genes involved in the plant’s hormonal response to salt or drought [65]. Silicon has been shown to act as an up-regulator of abscisic acid biosynthesis in rice and as a down-regulator of jasmonic acid biosynthesis in rice and certain *Leguminosae* species [66,67]. Additionally, [68] provided a comprehensive list of housekeeping genes that are either up- or down-regulated in rice following Si supplementation under abiotic stress conditions. It has also been suggested that Si have an impact on gene expression levels leading to alleviating the negative effect of many other abiotic stresses, i.e., heavy metal pollution, via regulation of peroxidases and superoxide dismutase (SOD) genes, oxidative stress caused by reactive oxygen species (SOD and MAPK), low or high-temperature exposure—by ascorbic peroxidase (APX) and guaiacol peroxidase (GPOX) genes [18,69].

Generally, understanding Si at the molecular level has revealed its pivotal role in maintaining plant homeostasis under abiotic stress conditions. Notably, Si modulates gene expression through two main mechanisms: (1) activating the transcription of genes that encode various osmo-protective proteins like dehydrins or aquaporins [26,70,71,72], and (2) regulating gene expression via intricate intracellular signaling pathways that involve plant hormones and MAPK kinases [65,73,74]. While the precise role of silicon in these processes remains unclear and little is known about Si-regulated molecular mechanisms in forest tree species like European beech, it is conceivable that the general gene expression pathway induced by silicon in plants follows a similar pattern. It has been shown that a biotic stress caused by *P. plurivora* attack increased the accumulation of Si in young oak leaves exposed to phosphorus deficiency [75].

### 4.3. Effect of Provenance and Si Supply on Plant Health

Silicon is considered a useful element for plants as its supply increases growth and yield, reduces susceptibility to pathogens and pests, and improves response to abiotic stress. The importance of silicon supply for plant production and mitigation of abiotic and biotic stresses is still poorly understood, so this study contributes to understanding the response of beech trees to silicon supplementation. Utilizing the positive effects of silicon will increase the quality of forest crops and thus the stability of future stands. In agricultural and horticultural crops, silicon helps uptake nutrients such as phosphorus and others, contributing to improved plant growth parameters and thus better quality and higher yield growth [76]. Thus, in the present study, tests were conducted with the silicon formulation OPTYSIL® on forest species. Beech seedlings of different provenances in Poland were used in the study to better reflect possible plant responses to the applied treatment.

The availability of water in the soil is necessary for the uptake of silicon by the plant; therefore, the silicon content in both leaves and roots was always higher in Spray + Water than in the other experimental treatments, e.g., in leaves, Spray + Water = 339.5 mg/kg and Spray + Drought = 228.5 mg/kg. In Drought, the Si content (178.9 mg/kg) was lower than in the irrigated beech seedlings (Control = 181.1 mg/kg).

In our study, we found (similar to observations made by Wang and Han in alfalfa cultivars, Maguire et al. [77] in sugar maple, and Turpault et al. [78] in European beech seedlings) that Si also accumulates in the roots, although most authors indicate higher silicon content in the transpiration organs like leaves and lower in the absorbent organs like roots of many crop plants, suggesting that Si deposition is influenced by the upward flow of the evapotranspiration stream [10,13,79,80]. Experiments with rice mutants in which the *Lsi*, silicon transporter genes encoding membrane proteins responsible for silicon uptake and transport in the plant, were silenced led to the identification of, among others, two genes, *Lsi*1 and *Lsi*2, in rice roots and the *Lsi*6 gene in the upper shoots of the plant. The activation of these genes occurs depending on the plant’s silicon requirement [13,81]. Currently, a series of *Lsi* genes located in both roots and upper shoots are known in *Arabidopsis thaliana* (L.), as well as in most crop plants such as *Zea mays* (L.), *Hordeum vulgare* (L.), and *Triticum aestivum* (L.). Understanding the mechanism of their regulated transcription could be highly beneficial in agricultural practice [82].

When *F. sylvatica* leaves were sprayed, the Si content in the roots appeared to be higher regardless of the treatment. The high silicon content of the control seedlings indicates that roots are generally the main site of Si allocation regardless of treatment. Presumably, the higher silicon content enables plants to better manage their water resources in the first years of growth. This could be important for surviving unfavorable periods of drought and increasing the efficiency of photosynthesis.

## 5. Conclusions

Our research has provided several important insights into the role of silicon in improving the drought tolerance in European beech seedlings. First, we found that silicon content in leaves increased more when soil moisture was higher compared with drought. Similarly, silicon accumulation in the roots was higher after foliar silicon treatment and consistent watering, compared with both the control group and drought conditions following silicon treatment. Interestingly, the silicon content in the roots was significantly higher than in the leaves under control conditions, but this difference was reduced after silicon application, suggesting that foliar silicon treatment helps balance silicon distribution between roots and shoots.

Furthermore, our findings indicate that beech seedlings are able to maintain relatively high PSII efficiency under moderate drought conditions, but their physiological responses are complex and influenced by several factors. The observed discrepancies in chlorophyll fluorescence parameters such as Fv/Fm and DIo/RC suggest a differential interplay between stress acclimation and potential photoprotective strategies. The results also highlight the potential for provenance-specific responses and the influence of treatment interactions over time, emphasizing the dynamic nature of beech seedlings responses to environmental challenges. This complexity suggests that while certain physiological metrics may indicate improved functioning under stress following Si application (even if physiological traits did not improve compared with the control treatment), they need to be interpreted in the broader context of overall plant condition and over time during the growing season.

In terms of gene expression, we found that the highest overall expression of *hsp*70 and *hsp*90 genes, which are associated with stress responses, occurred under drought conditions after silicon treatment. There were also notable differences in *hsp*70 expression between provenances, with the strongest response occurring in the Lesko, Zdroje, and Kielce provenances, while the Nawojowa provenance showed the weakest response. A similar pattern of regulation was observed for *hsp*90 expression, particularly in the Zdroje provenance.

These findings are set against the backdrop of dynamic and ongoing climatic changes affecting forests globally. The role of silicon in enhancing plant resilience is increasingly recognized, as emphasized by organizations such as the International Society for Silicon in Agriculture (ISSAG), which promotes knowledge sharing on the use of silicon in agriculture and related fields, including forestry. Our research contributes to this growing body of knowledge by exploring the impact of silicon on the physiological responses of European beech and the expression of stress-related genes such as *hsp*70 and *hsp*90. Our research highlights potential differences in the beneficial role of silicon application arising from the large differences in wild populations of forest tree species.

## Figures and Tables

**Table 1:** Multifactor ANOVA for chlorophyll fluorescence parameters and results of post-hoc HSD Tukey test. The same letters denote lack of significant differences at the 0.05 level of significance; Jul—July, Oct—October.

		Fo/Fm		Fv/Fm		PI Inst.		ABS/RC		DIo/RC		TRo/RC		ETo/RC		PI Abs		PI Total	
**ANOVA Effects**		**F**	***p***	**F**	***p***	**F**	***p***	**F**	***p***	**F**	***p***	**F**	***p***	**F**	***p***	**F**	***p***	**F**	***p***
Provenance		3.13	0.027	3.18	0.025	0.14	0.938	0.71	0.545	3.15	0.026	0.15	0.93	2.34	0.074	0.14	0.938	0.4	0.754
Treatment		1.91	0.129	1.9	0.131	0.86	0.464	8.91	<0.001	6.73	<0.001	7.52	<0.001	34.52	<0.001	0.86	0.463	0.72	0.542
Date		135.52	<0.001	135.3	<0.001	86.99	<0.001	5.42	0.021	42.23	<0.001	40.11	<0.001	198.14	<0.001	87	<0.001	130.01	<0.001
Prov. × Treatment		1.94	0.048	1.93	0.049	1.32	0.226	3.72	<0.001	4.06	<0.001	2.74	0.005	1.28	0.251	1.32	0.227	0.9	0.526
Prov.*Date		1.38	0.251	1.43	0.235	2.04	0.110	1.1	0.349	2.16	0.094	0.54	0.658	2.82	0.04	2.04	0.11	1.64	0.181
Treatment*Date		9.83	<0.001	9.77	<0.001	2.51	0.059	3.29	0.022	9.46	<0.001	1.12	0.34	11.83	<0.001	2.51	0.059	3.4	0.019
Prov.*Treatment*Date		3.39	0.001	3.39	0.001	1.66	0.100	2.32	0.016	4.23	<0.001	1.41	0.183	0.98	0.455	1.66	0.101	0.58	0.815
HSD Tukey test		Mean	HSD	Mean	HSD	Mean	HSD	Mean	HSD	Mean	HSD	Mean	HSD	Mean	HSD	Mean	HSD	Mean	HSD
Control	Jul	0.19	d	0.81	a	2.54	a	2.19	a	0.42	cd	1.77	ab	1.07	a	3.05	a	1.24	a
Drought	Jul	0.2	c	0.8	b	2.22	bc	2.13	ab	0.43	bcd	1.7	c	0.98	d	2.66	bc	1.02	b
Spray + D	Jul	0.19	cd	0.81	ab	2.43	ab	2.2	a	0.43	bcd	1.78	a	1.06	ab	2.92	ab	1.23	a
Spray + W	Jul	0.19	cd	0.81	ab	2.3	ab	2.2	a	0.43	bcd	1.77	ab	1.03	bc	2.75	ab	0.99	b
Control	Oct	0.23	a	0.77	d	1.68	cd	2.24	a	0.52	a	1.71	abc	0.99	cd	2.01	cd	0.54	c
Drought	Oct	0.21	bcd	0.79	abc	1.74	cd	1.98	b	0.41	d	1.57	d	0.8	e	2.08	cd	0.62	c
Spray + D	Oct	0.23	ab	0.77	cd	1.44	d	2.13	ab	0.48	ab	1.65	bcd	0.85	e	1.73	d	0.47	c
Spray + W	Oct	0.22	ab	0.78	cd	1.89	bcd	2.12	ab	0.48	abc	1.64	cd	0.94	d	2.27	bcd	0.61	c

## Data Availability

Data are contained within the article.
